# Frequency, Risk Factors, and Prognosis of Dehydration in Acute Stroke

**DOI:** 10.3389/fneur.2019.00305

**Published:** 2019-03-29

**Authors:** Elena Cortés-Vicente, Daniel Guisado-Alonso, Raquel Delgado-Mederos, Pol Camps-Renom, Luis Prats-Sánchez, Alejandro Martínez-Domeño, Joan Martí-Fàbregas

**Affiliations:** Department of Neurology, Hospital de la Santa Creu i Sant Pau, Biomedical Research Institute Sant Pau (IIB Sant Pau), Universitat Autònoma de Barcelona, Barcelona, Spain

**Keywords:** stroke, dehydration, urea, creatinine, prognosis, risk factors

## Abstract

**Objective:** To determine the frequency, risk factors, and impact on the outcome of dehydration after stroke.

**Methods:** In this cross-sectional observational study, we included prospectively and consecutively patients with ischemic and hemorrhagic stroke. The serum Urea/Creatinine ratio (U/C) was calculated at admission and 3 days after the stroke. Dehydration was defined as U/C>80. Patients were treated in accordance with the standard local hydration protocol. Demographic and clinical data were collected. Neurological severity was evaluated at admission according to the NIHSS score; functional outcome was assessed with the modified Rankin scale score (mRS) at discharge and 3 months after the stroke. Unfavorable outcome was defined as mRS > 2.

**Results:** We evaluated 203 patients; 78.8% presented an ischemic stroke and 21.2% a hemorrhagic stroke. The mean age was 73.4 years ±12.9; 51.7% were men. Dehydration was detected in 18 patients (8.9%), nine patients at admission (4.5%), and nine patients (4.5%) at 3 days after the stroke. Female sex (OR 3.62, 95%CI 1.13–11.58, *p* = 0.03) and older age (OR 1.05, 95%CI 1–1.11, *p* = 0.048) were associated with a higher risk of dehydration. Dehydration was significantly associated with an unfavorable outcome at discharge (OR 5.16, 95%CI 1.45–18.25, *p* = 0.011), but the association was not significant at 3 months (OR 2.95, 95%CI 0.83–10.48, *p* = 0.095).

**Conclusion:** Dehydration is a treatable risk factor of a poor functional outcome after stroke that is present in 9% of patients. Females and elders present a higher risk of dehydration.

## Introduction

The clinical guidelines state that dehydration after stroke results in a poorer vital and functional prognosis (1–4). Dehydration increases hemoconcentration and blood viscosity and decreases blood pressure, factors that may worsen the effects of brain ischemia (5), resulting in greater brain damage, and more severe symptoms. Dehydration is related also with a higher risk of complications, such as venous thrombosis. Among other factors, stroke patients may be predisposed to dehydration because of decreased oral intake of water due to dysphagia (6) or low level of consciousness. Stroke occurs more frequently in the elderly, and dehydration is common in these patients (7). Dehydration is important also because of its economic impact, as dehydration at admission is associated with higher admission costs in acute ischemic stroke (8).

There is substantial variation in hydration status definition and diagnostic approach to dehydration (9). The blood Urea/Creatinine ratio (U/C) (10, 11), the blood urea nitrogen/Creatinine ratio (BUN/C) (6, 8, 12) and plasma osmolality (13) have been used to detect dehydration. Multi-frequency bioelectrical impedance has also been tested, but appeared ineffective to diagnose dehydration correctly after stroke (14). These studies suggested that dehydration after stroke is a prevalent phenomenon, with a frequency around 60% measured with de U/C ratio (10) and around 53% with the BUN/C ratio (6), and that it is associated with a poor prognosis (8, 10, 12, 13). However, these studies are retrospective and did not include a follow-up evaluation. Thus, it is still unclear if dehydration significantly influences outcome.

Our aim was to determine the frequency and risk factors of dehydration after acute stroke, and its effects on the outcome at discharge and 3 months after stroke, using a blood biomarker of dehydration as a diagnostic tool.

## Materials and Methods

### Patients

This is a cross-sectional observational study of patients prospectively and consecutively included during a 10-month period. This period of time was estimated to include 185 patients, the sample size needed for an estimated proportion of 62% (10), a 95% confidence level, and a desired precision of 7%. All patients had an ischemic or hemorrhagic stroke, confirmed by neuroimaging techniques (CT or MRI). Patients under 18 years old, admitted >72 h after stroke onset and with a previous modified Rankin scale score (mRS) >2 were excluded.

### Clinical Evaluation

Patients were evaluated by a neurologist at admission, at discharge and 3 months after stroke. Assessment included evaluation of stroke severity according to the National Institute of Health Stroke Scale (NIHSS) score and functional evaluation according to the mRS score. Investigators were blinded to the U/C ratio results. Unfavorable outcome was defined as mRS > 2. The functional evaluation of the patients was assessed during face-to-face interviews 3 months after stroke. If a face-to-face interview was not possible, the data were obtained by a phone interview. Responses of patients, relatives or caregivers were recorded. Demographic data (age, sex) and functional status before the stroke according to the mRS score were collected. The main clinical features of the disease, such as stroke type (ischemic or hemorrhagic), arterial territory if ischemic stroke, stroke etiology according to TOAST classification (15) if ischemic stroke, time elapsed from stroke onset to admission, reperfusion therapies if ischemic stroke, admission to the Stroke Unit, presence of dysphagia, presence of aphasia, length of hospitalization in days, and cardiovascular risk factors were also recorded. History of potential risk factors of dehydration, such as heart or renal failure, treatment with diuretics, living alone, vomiting, diarrhea, or fever, were recorded also.

### Study Protocol

Patients were treated according to the standard local protocol: nil per os during the first 24 h from the onset of the stroke and intravenous hydration with saline serum (500 ml/6 h) with potassium supplements, adjusted in selected cases when needed (heart or renal failure history, presence of hypoglycaemia, vomiting, diarrhea, or fever). Glucose levels were monitored periodically. After a standardized swallowing test conducted by a trained nurse, patients started oral hydration, and nutrition (or by nasogastric tube if severe dysphagia) from day 2 onwards. Blood samples were obtained at admission and 3 days after the stroke. U/C ratio was calculated. Dehydration was defined as a blood U/C ratio >80 (10). The study was approved by the ethics committee of Hospital de la Santa Creu i Sant Pau. Verbal informed consent was obtained from all of the patients or their legal representatives. A signed consent was not deemed necessary by the committee due to the absence of a change in the routine management of the patients and also that the data obtained were anonymous.

### Statistical Analysis

A descriptive data analysis was performed. Demographic characteristics were reported as means and standard deviations or percentages. NIHSS scores are reported as medians and interquartile ranges. Differences in baseline characteristics between dehydrated and non-dehydrated patients were compared using a chi-square (χ2) test for categorical variables and a *t*-Test analysis was used to compare quantitative variables. A significant difference was defined as *p* < 0.05. Univariate logistic regression models between the dependent variable (presence of dehydration) and sex, age, subtype of stroke, NIHSS score at admission, time from stroke onset to evaluation, presence of heart, and renal failure, previous use of diuretics, presence of vomiting, fever and diarrhea, presence of dysphagia and aphasia, admission at the Stroke Unit and living alone, were investigated. In another analysis, univariate logistic regression models were used also between the dependent variable (unfavorable prognosis) and dehydration, NIHSS score at admission, sex, age, subtype of stroke and admission at the Stroke Unit, at discharge and 3 months after the stroke. A multivariate logistic regression method was used to ascertain independent associations between those factors with *p* < 0.10 from the univariate analysis. Data analysis was carried out using Stata 13.0 (StataCorp, College Station, TX) for Windows.

## Results

We studied 203 patients; 160 (78.8%) with ischemic stroke and 43 (21.2%) with hemorrhagic stroke. Their mean age was 73.4 years (SD 12.9); 51.7% of them were men. The median NIHSS at admission was five (IQR 2–14). Patients were admitted a mean of 9.8 (SD 14) hours after the stroke onset. All of the patients were hydrated initially intravenously within the first 24 h of admission. From the second day on, 168 (82.8%) were hydrated orally and 35 (17.2%) needed hydration by nasogastric tube because of severe dysphagia or low level of consciousness. All of the included patients were evaluated at 3 months, 164 during face-to-face interviews, and 39 by phone.

Dehydration based on the blood U/C ratio was detected in 18 patients (8.9%) at some point during the hospitalization. Nine patients (4.5%) were dehydrated at admission and, after 3 days follow-up, the U/C ratio was normal in eight patients and only one remained dehydrated. The other nine patients (4.5%) were not dehydrated at admission, but were dehydrated 3 days after the stroke.

Patients who presented dehydration were older (*p* = 0.019), more often women (*p* = 0.009) and with a higher frequency of dysphagia (*p* = 0.026) compared to non-dehydrated patients. No differences in other variables were found (Table 1). From the multivariable logistic regression analysis, the variables associated with dehydration were female sex (OR 3.62, 95% CI 1.13–11.58, *p* = 0.03) and older age (OR 1.05, 95% IC 1–1.11, *p* = 0.048).

**Table 1:** Comparison of clinical features in patients classified as either dehydrated or non-dehydrated during hospital admission and *p*-values from statistical analysis.

	**Dehydrated patients (*n* = 18)**	**Hydrated patients (*n* = 185)**	***p* value**
Age, years. Mean (SD)	80.2 (9.5)	72.8 (13.0)	**0.019**
Sex (Female/Male) (% Males)	14/4 (22.2)	84/101 (54.6)	**0.009**
Stroke type (Ischemic/Hemorrhagic) (% Ischemic)	17/1 (94.4)	143/42 (77.3)	0.089
TOAST classification	(*n* = 17)	(*n* = 143)	0.822
Large-artery atherosclerosis (%)	3 (17.6)	21(14.7)	
Cardioembolism (%)	7 (41.2)	51 (35.7)	
Small-vessel occlusion (%)	0 (0)	11 (7.7)	
Stroke of other determined etiology (%)	1 (5.9)	9 (6.3)	
Stroke of undetermined etiology (%)	6 (35.3)	51 (35.7)	
Anterior arterial territory *n*, (%)	13/17 (76.5)	120/143 (83.9)	0.531
NIHSS^a^ at admission (median–IQR)	10 (3–13)	5 (2–14)	0.346
Hours from onset. Mean (SD)	7.2 (6.4)	10.1 (14.5)	0.395
Renal failure *n*, (%)	1/18 (5.6)	26/185 (14.1)	0.311
Heart failure *n*, (%)	4/18 (22.2)	21/185 (11.4)	0.180
Dysphagia *n*, (%)	7/18 (38.9)	32/185 (17.3)	**0.026**
Aphasia *n*, (%)	4/18 (22.2)	60/185 (32.4)	0.373
Diuretic treatment *n*, (%)	8/18 (44.4%)	72/185 (38.9)	0.647
Reperfusion therapy *n*, (%)	3/17 (17.7%)	28/143 (19.6)	0.849
Stroke Unit admission *n*, (%)	10/18 (55.6)	132/185 (71.4)	0.163
Arterial hypertension (%)	12/18 (66.7)	126/185 (68.1)	0.900
Diabetes Mellitus *n*, (%)	4/18 (22.2)	41/185 (22.2)	0.995
Vomiting *n*, (%)	2/18 (11.1)	14/185 (7.6)	0.594
Diarrhoea *n*, (%)	0/18 (0)	3/185 (1.6)	0.586
Fever *n*, (%)	3/18 (16.7)	14/185 (7.6)	0.183
mRS^b^ > 2 at discharge *n*, (%)	14/18 (77.8)	80/185 (43.2)	**0.005**
mRS > 2 at 3 months *n*, (%)	13/18 (72.2)	80/185 (43.2)	**0.018**
Length of hospitalization, days. Mean (SD)	16.4 (9.9)	12.6 (9.4)	0.098
Living alone *n*, (%)	7/18 (38.9)	49/185 (26.5)	0.261

a *National Institute of Health Stroke Scale*.

b *Modified Rankin scale score*.

**Figure 1:**
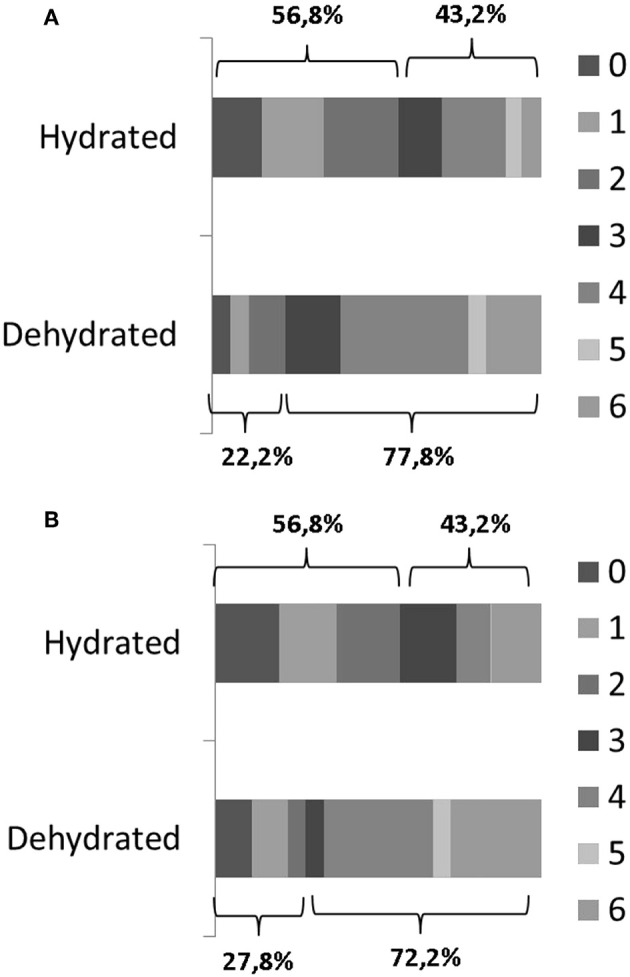
Comparison of mRS scores in dehydrated patients (*n* = 18) and non-dehydrated patients (*n* = 185) at discharge **(A)** and after 3 months **(B)**.

Patients who presented an unfavorable outcome at discharge were older (*p* = 0.024), had a higher NIHSS score at admission (*p* < 0.0001), had a higher frequency of dehydration (*p* = 0.005), and a non-significant trend to be females (*p* = 0.062) (Table 2). From the multivariable logistic regression analysis, the variables associated with an unfavorable outcome were NIHSS score (OR 1.2, 95% CI 1.14–1.27, *p* < 0.0001) and dehydration (OR 5.16, 95% CI 1.45–18.25, *p* = 0.011) (Figure 1A).

**Table 2:** Comparison of clinical features in patients with good prognosis (mRS ≤ 2) and poor prognosis (mRS > 2) at discharge and *p*-values from statistical analysis.

	**mRS^a^ ≤ 2 (*n* = 109)**	**mRS > 2 (*n* = 94)**	***p*-value**
Age, years. Mean (SD)	71.0 (12.9)	75.1 (12.6)	**0.024**
Sex (Female/Male) (% males)	46/63 (57.8%)	52/42 (44.7%)	0.062
NIHSS^b^ at admission (median–IQR)	3 (1–6)	13 (5–18)	**<0.0001**
Dehydration *n*, (%)	4/105 (3.7)	14/80 (14.9)	**0.005**
Stroke type (Ischemic/Hemorrhagic) (% Ischemic)	89/20 (81.7)	71/23 (75.5)	0.287
Admission at Stroke Unit *n*, (%)	75/34 (68.8)	67/27 (71.3)	0.702

a *Modified Rankin scale score*.

b *National Institute of Health Stroke Scale*.

Similarly, patients who presented an unfavorable outcome 3 months after the stroke were significantly older (*p* = 0.001), had a higher NIHSS score at admission (*p* < 0.001), were more often females (*p* = 0.045), and were more often dehydrated (*p* = 0.018) (Table 3). From the logistic regression analysis, the variables associated with an unfavorable outcome were high NIHSS score (OR 1.24, 95% CI 1.16–1.32, *p* < 0.0001) and older age (OR 1.04, 95% IC 1.01–1.07, *p* = 0.012). Dehydration was not significantly associated with the outcome at 3 months (OR 2.95, 95% CI 0.83–10.48, *p* = 0.095) (Figure 1B).

**Table 3:** Comparison of clinical features in patients with good prognosis (mRS ≤ 2) and poor prognosis (mRS > 2) 3 months after the stroke and *p*-values from statistical analysis.

	**mRS^a^ ≤ 2 (*n* = 110)**	**mRS > 2 (*n* = 93)**	***p*-value**
Age, years. Mean (SD)	70.1 (SD 13.4)	76.3 (SD 11.5)	**0.001**
Sex (Female/Male) (% males)	46/64 (58.2)	52/41 (44.1)	**0.045**
NIHSS^b^ at admission (median–IQR)	3 (1–5)	13 (6–18)	**<0.0001**
Dehydration *n*, (%)	5/105 (4.5)	13/80 (14.0)	**0.018**
Stroke type (Ischemic/Hemorrhagic) (% Ischemic)	91/19 (82.7)	69/24 (74.2)	0.138
Admission at Stroke Unit *n*, (%)	75/35 (68.2)	67/26 (72.0)	0.550

a *Modified Rankin scale score*.

b *National Institute of Health Stroke Scale*.

## Discussion

In our study, 8.9% of the patients presented dehydration based on the U/C ratio at any point during the 3 first days of hospitalization. Our data revealed that dehydration is associated with a 5-fold higher risk of poor outcome at hospital discharge and is related to female sex and older age.

Our data regarding dehydration frequency (8.9%) showed a notable difference when compared with the only other study that used the U/C ratio, in which 62% of patients presented dehydration (10). Other studies using other parameters detected also a higher frequency (53%) of dehydration (6). Differences in prevalence of dehydration risk factors between both populations may explain these discrepancies. Also differences in the hydration protocol used in different hospitals may be the cause, with a lower frequency in our patients because of the standardized protocol we use. The higher number of blood tests that were used in the reference study (10) (a median of four tests per patient compared to two systematic blood controls in our study), may have increased the possibility of detecting dehydration. The reference study included a large number of patients, but its design was retrospective, the only functional evaluation that was reported was at discharge, and a detailed description of the hydration protocol they used was not provided.

Previous studies have associated elder age and female sex to dehydration (10, 16, 17). Older adults have less body water, reduced thirst signals that may lead to less water intake, and also the function of the kidneys is reduced with age and they may have problems with urine concentration. Women also have lesser body water than men. In our series, patients who were dehydrated were more likely to be elders and females. We found that dysphagia was more frequent in patients presenting dehydration, but our data did not proved it as an independent risk factor of dehydration by the logistic regression analysis. In the literature, we found discrepant results regarding dysphagia as a dehydration risk factor (6, 18).

Urea and Creatinine assays are rapid, cheap, and easily available to diagnose and follow up dehydration and are included in the routine assessment of every patient with stroke. However, we acknowledge that the U/C ratio may be inaccurate in certain medical conditions such as renal or cardiac disease, gastrointestinal bleeding, diarrhoea, vomiting, infections, and use of diuretics (10, 11). In our sample, we found no significant differences in these conditions between dehydrated and non-dehydrated patients. Thus, the main limitation of our study is the absence of a “gold standard” for measuring dehydration. However, other blood biomarkers that have been proposed, such as BUN/C ratio (6, 8, 12) or plasma osmolality (13), have also similar limitations and are more difficult to obtain in the Emergency Department. Multi/frequency bioelectrical impedance is ineffective and additionally it is not easily available (14). Although of help, clinical data such as dry mouth, dry skin, thirst or dark, and scarce urine are subjective parameters. Taking these facts into consideration, we think that the U/C ratio is the easiest, most cost-effective, and most reliable biomarker of hydration to use in stroke patients. Therefore, we recommend these assays at the Stroke Unit (19) to improve the maintenance of homeostasis of patients with stroke.

Another limitation of the study is that although we included the statistically necessary number of patients, the group of patients with dehydration is relatively small. We expected a higher frequency of dehydration, based in previous studies. With a larger sample of patients it is likely that we would have found a significant association between outcome at 3 months and dehydration and we also would have been able to analyse separately risk factors associated with dehydration at admission and dehydration during hospitalization.

However, the strength of our study is that we included consecutive evaluation of a large number of patients at discharge and 3 months after the stroke. This provides consistency and reliability to our study. The findings of our study are important because dehydration is a potentially preventable and easily treatable complication in stroke. According to our results, elders and women should be carefully evaluated because they are at a higher risk of dehydration after stroke.

Although dehydration is an important prognostic risk factor in stroke patients, we are not certain that treating dehydration significantly improves outcome. A study showed that BUN/C ratio-based saline hydration therapy in patients with acute ischemic lacunar stroke significantly increased the rate of favorable clinical outcome with functional independence at 3 months after stroke. However, this was a non-blinded study that used historical controls (20). So, further studies are required to determine whether reversing dehydration after stroke significantly improves the functional outcome.

In conclusion, dehydration is a common risk factor associated with a poor functional outcome after stroke. Dehydration should be systematically studied using serum biomarkers, especially in elders and females.

## Data Availability

All datasets generated for this study are included in the manuscript.

## Author Contributions

EC-V and JM-F contributed conception and design of the study. EC-V and DG-A contributed acquisition of data. EC-V organized the database and performed the statistical analysis, wrote the first draft of the manuscript. All authors contributed interpretation of data, manuscript revision, read, and approved the submitted version.

### Conflict of Interest Statement

The authors declare that the research was conducted in the absence of any commercial or financial relationships that could be construed as a potential conflict of interest. The reviewer XU declared past co-authorships with one of the authors JM-F to the handling Editor.
