# Corrigendum: Frequency, Risk Factors, and Prognosis of Dehydration in Acute Stroke

**DOI:** 10.3389/fneur.2020.00717

**Published:** 2020-07-17

**Authors:** Elena Cortés-Vicente, Daniel Guisado-Alonso, Raquel Delgado-Mederos, Pol Camps-Renom, Luis Prats-Sánchez, Alejandro Martínez-Domeño, Joan Martí-Fàbregas

**Affiliations:** Department of Neurology, Hospital de la Santa Creu i Sant Pau, Biomedical Research Institute Sant Pau (IIB Sant Pau), Universitat Autònoma de Barcelona, Barcelona, Spain

**Keywords:** stroke, dehydration, urea, creatinine, prognosis, risk factors

There is an error in the Funding statement. The correct number for **Instituto de Salud Carlos III, RETICS (Redes Temáticas de Investigación Cooperativa en Salud) INVICTUS PLUS and FEDER** is RD06/0019/0010. The corrected Funding statement appears below.

There is an error in the Funding statement. The correct Name for the Funder is **Instituto de Salud Carlos III, RETICS (Redes Temáticas de Investigación Cooperativa en Salud) INVICTUS PLUS and FEDER**. The corrected Funding statement appears below.

The authors apologize for this error and state that this does not change the scientific conclusions of the article in any way. The original article has been updated.

